# Are suspensory ligaments important for middle ear reconstruction?

**DOI:** 10.1371/journal.pone.0255821

**Published:** 2021-08-24

**Authors:** Eileen Y. Brister, Robert H. Withnell, Pavel Shevchenko, Claus-Peter Richter

**Affiliations:** 1 Department of Otolaryngology, Northwestern University Feinberg School of Medicine, Chicago, Illinois, United States of America; 2 Department of Speech, Language, and Hearing Sciences, Indiana University, Bloomington, Indiana, United States of America; 3 Advanced Photon Source, Argonne National Lab, Lemont, Illinois, United States of America; 4 Department of Biomedical Engineering, Northwestern University, Evanston, Illinois, United States of America; 5 Department of Communication Sciences and Disorders, Northwestern University, Evanston, Illinois, United States of America; 6 The Hugh Knowles Center, Northwestern University, Evanston, Illinois, United States of America; Universidade Federal de Sao Paulo/Escola Paulista de Medicina (Unifesp/epm), BRAZIL

## Abstract

As the resolution of 3D printing techniques improves, the possibility of individualized, 3-ossicle constructions adds a new dimension to middle ear prostheses. In order to optimize these designs, it is essential to understand how the ossicles and ligaments work together to transmit sound, and thus how ligaments should be replicated in a middle ear reconstruction. The middle ear ligaments are thought to play a significant role in maintaining the position of the ossicles and constraining axis of rotation. Paradoxically, investigations of the role of ligaments to date have shown very little impact on middle ear sound transmission. We explored the role of the two attachments in the gerbil middle ear analogous to human ligaments, the posterior incudal ligament and the anterior mallear process, severing both attachments and measuring change in hearing sensitivity. The impact of severing the attachments on the position of the ossicular chain was visualized using synchrotron microtomography imaging of the middle ear. In contrast to previous studies, a threshold change on the order of 20 dB across a wide range of frequencies was found when both ligaments were severed. Concomitantly, a shift in position of the ossicles was observed from the x-ray imaging and 3D renderings of the ossicular chain. These findings contrast with previous studies, demonstrating that these ligaments play a significant role in the transmission of sound through the middle ear. It appears that both mallear and incudal ligaments must be severed in order to impair sound transmission. The results of this study have significance for middle ear reconstructive surgery and the design of 3D-printed three-ossicle biocompatible prostheses.

## Introduction

As 3D printing techniques improve in ability to print biocompatible materials at a high resolution, the possibility of designing a three-ossicle middle ear prosthesis individualized to the ear of a patient becomes more achievable [1,2]. While these designs may have potential to increase hearing outcomes by more closely simulating a healthy ossicular chain, they also introduce a higher level of complexity and design challenges. In the healthy ear, ligaments connect the ossicles to the cochlea and to the walls of the middle ear cavity. Understanding how these ligaments contribute to sound transmission in a normal ear is essential to designing 3-ossicle prostheses that incorporate ligament structures to optimize hearing outcomes in reconstructed ears.

The functional role of the middle ear ligaments has long been described as suspending the ossicles [3–8], limiting the direction of motion, and defining the rotational axis [9–12]. However, published experimental data does not support this putative role, with no substantial change to the middle ear transfer function observed when selected ligaments are removed from the system [6,13,14].

The posterior incudal ligament and the anterior mallear ligament, or a thin, bony analogue, are the two major attachments consistently present across most mammalian ears [10,15,16]. These are also the two attachments widely discussed as defining the axis of rotation for the ossicular chain, particularly at low frequencies [9–12]. Published experimental results only report minimal impacts on sound transmission from the experimental severing of one of these ligaments [6,13,14], with the effect of severing both together remaining unknown.

The gerbil only has two attachments analogous to the ligaments found in the middle ears of humans and other mammals: the posterior incudal ligament, and the anterior mallear process. The anterior mallear process is not a true ligament, but rather a very thin, translucent, bony process analogous to the human anterior mallear ligament [10,12,17]. This smaller number of attachments, specifically the two attachments purported to define axis of rotation [9–12], simplifies the investigation of the role of middle ear attachments compared to the human middle ear, the experimental model used in previous studies [13,14]. The human middle ear is a challenging model for examining the role of ligaments, with disagreement about the exact number of ligaments [8,18,19], and small ligaments may be mistaken for mucosal folds and vice versa [18].

Mathematical models of the middle ear that include ligaments typically represent them as springs, and simulate severing of ligaments by reducing the spring constant [13,20,21]. Some have been paired with supporting experimental evidence with mallear ligaments severed and minimal change in threshold predicted by modelling and experimentally observed [13]. For nonhuman models and those predicting severing of all ligaments and analogs [20], experimental evidence validating the model is not presented.

Our goal is to experimentally evaluate the function of both mallear and incudal ligaments together. This paper examines the impact of severing the anterior mallear process and the posterior incudal ligament of the gerbil middle ear on sound transmission and ossicular chain position. The two attachments were severed using a surgical laser. The impact of severing these ligaments on sound transmission was quantified by measuring the change in hearing thresholds with compound action potential measurements in response to both bone conduction and air conduction stimulation. We hypothesize that severing both attachments will result in a conductive hearing loss, with an elevation in air conduction thresholds, no change in bone conduction thresholds, and a shift in the position of the ossicles.

## Methods

### Ethics statement

Care and use of animals were conducted following the guidelines in the National Institutes of Health Guide for Care and Use of Laboratory Animals, and all animal procedures were approved by the Animal Care and Use Committee of Northwestern University. Gerbils originated from our own colony, maintained at the animal facility at Northwestern University. Animals were housed in groups of up to four animals; food and water were provided ad libitum; enrichments, nesting materials, and shelters were given.

### Animal model and procedure outline

Twenty-four adult gerbils (*Meriones unguiculatus*) of either sex, weighing 65–105 g, were used in this study. Surgery was performed to access and open the bulla, visualize and sever the anterior mallear process (AMP), posterior incudal ligament (PIL), and visualize the round window to place an electrode in the round window niche to measure compound action potentials (CAPs). The sequence of procedures was as follows: anesthetize the animal, access the bulla, open the bulla and expose the cochlea and place a round window electrode, create a second opening in the bulla to access the AMP and PIL, reseal the openings in the bulla, measure baseline CAP thresholds for air and bone conduction, sever the AMP or PIL, or both, measure the CAP thresholds for air and bone conduction, cut the other ligament (PIL or AMP), measure CAP thresholds for air and bone conduction, euthanize the animal and harvest temporal bones for x-ray imaging.

### Anesthesia

Anesthesia was induced with a mixture of ketamine (80–100 mg/kg) and xylazine (2–10 mg/kg) injected intraperitoneally and was maintained during surgery with isoflurane (1.0–3.0%) given via a nose cone. After surgery, anesthesia was maintained by 0.5–2.0% isoflurane in oxygen and nitrous oxide (50%). Vitals, including blood oxygen level, and respiratory rate, were monitored and logged in 15-minute intervals. The level of anesthesia was assessed by the toe pinch response.

### Surgery

The left ear was used for all experiments. The bulla was accessed through a retroauricular c-shaped skin incision and removal of muscles attached to the bulla via blunt dissection. After cutting the cartilaginous outer ear canal and removing the pinna, a small tube with an inner diameter of 1.45 mm coupled the speaker’s (Beyerdynamic DT770 Pro) speculum to the ear canal for air conduction stimulation.

Through an approximately 1mm x 1 mm opening in the bulla, caudal to the ear canal, a 125-μm diameter silver wire was hooked on the bony rim adjacent to the round window for CAP recordings. A small piece of tissue moistened with Ringer’s Lactated Solution was placed over the opening to reseal the bulla. A small remaining hole in the bulla allowed ventilation of the middle ear, preventing retraction of the tympanic membrane during recordings.

**Fig 1:**
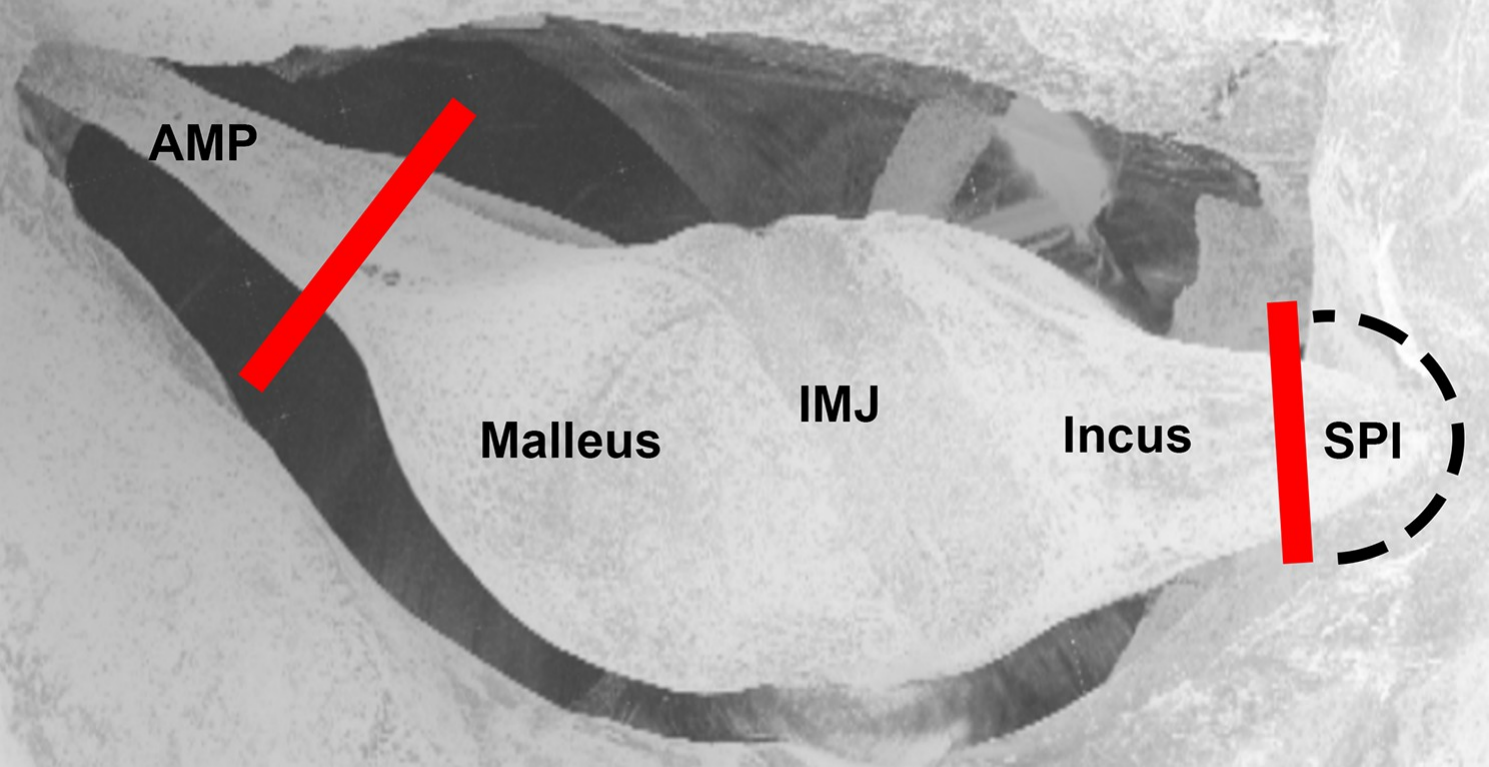
3D rendering of a microtomography image of the middle ear, positioned to approximate the coronal view of the ossicles from the surgical opening. The incus, malleus, anterior mallear process (AMP), short process of the incus (SPI), and incudomallear joint (IMJ) are visible. The dashed black line traces the approximate path of the posterior incudal ligament, which wraps around the tip of the short process and is largely hidden from view by a bony shelf. Solid red lines demarcate the locations where laser cuts were made to sever the anterior mallear process and posterior incudal ligament.

A second opening was made through the roof of the middle ear. The anterior mallear process and posterior incudal ligament were visible through this opening (Fig 1). The bulla was resealed by a small piece of moistened tissue paper.

The bone conduction transducer used in this experiment was a rectangular prism with a 21.5- x 14-mm surface, a 7.9 mm height, and a 9.6 g weight. The surface area was too large in relation to the size of the head of the gerbil for it to be consistently coupled. Therefore, a small screw was attached to the surface of the bone conduction transducer, creating a small contact surface. To couple this smaller surface to the gerbil’s head, the skull was exposed in the region superior to the access hole used for ligament severing by dissecting skin and muscles and removing the periosteum. Then, a 1/8 inch cube neodymium rare earth magnet (Eclipse N427) was glued to the skull. The bone conduction transducer could then be coupled to the gerbil’s skull through the screw attached to the transducer being magnetically attached to the magnet glued to the skull. This provided a consistent coupling mechanism that was easily detached during subsequent ligament severing and air conduction testing.

### Sectioning of the suspensory attachments

The anterior mallear process was severed using a surgical CO_2_ laser (Weinschel, Frederick, MD) coupled to an Oto-laserfiber (Omniguide, Lexington, MA). The laser was operated in single pulse mode with 100 ms pulse length and 4 Watt power setting. Charred remains of the process were manually cleared, and the severing of the attachment was confirmed using a small, custom-made hook made from 25 μm diameter tungsten wire glued to the inside of a 30 G needle. To sever the posterior incudal ligament, the tip of the short process of the incus connecting to the posterior incudal ligament was removed with the CO_2_ laser at a 5.0 Watt power setting. Charred remains of the connection were manually cleared with the hook, and severing was visually confirmed. The AMP was severed before the PIL in seven ears, the PIL was severed before the AMP in seven ears, and both attachments were severed simultaneously in three ears.

Ears were included in the analysis if severing of processes was verified, either through imaging or by sweeping the aforementioned hook around the region where attachments were severed.

### Acoustic stimuli

Pure tone bursts (24 ms long including a 2 ms rise/fall time and a 20 ms plateau time) were generated by custom-written software (TestPoint^TM^, Capital Equipment Corp., Billerica, MA). The amplitude of the computer-generated signal was controlled by an attenuator. It was used to drive a Beyerdynamic DT770 Pro headphone speaker (Beyerdynamic GMBH & Co. KG Heilbronn, Germany) via an audio amplifier (RA 150 amplifier, Alesis, Cumberland, RI). For each condition, the carrier frequency of the tone burst was changed systematically from 32,000 to 500 Hz, in one-octave steps in descending order.

For bone conduction threshold measurements, stimuli were the same as used for air conduction, except for the frequency range. The carrier frequency was presented in descending order from 16,000 to 500Hz in one-octave steps. Instead of the headphone speaker, a bone conduction transducer (Adafruit, New York, NY) was used.

### Calibration

To calibrate the Beyerdynamic DT770 Pro, it was coupled to a Brüel & Kjær 1/8 inch microphone using the same tube used to couple the speaker to the gerbil ear canal during experiments. The speaker output was measured in dB SPL at 0.1 V rms, 20 dB below the maximum level to ensure it was within the speaker’s linear response range.

Thresholds for CAPs in response to bone conduction stimuli were reported in dB attenuation based on the transducer’s input voltage. The goal of measuring bone conduction thresholds was to monitor changes in hearing via bone conduction across conditions. The purpose of the inclusion of bone conduction thresholds was to rule out the possibility of a systematic sensorineural hearing loss, with stable bone conduction thresholds indicating that any shifts in air conduction thresholds result from middle ear damage rather than cochlear or neural damage.

### Compound action potentials

Electrical cochlear responses during acoustic (air conduction) and mechanical (bone conduction) stimulation were recorded with the round window electrode (reference: neck muscles, ground: back muscles), filtered (300–3000 Hz, 12 dB/octave), and amplified (60 dB) using an ISO80 preamplifier (World Precision Instruments, Sarasota FL). The sampling rate was 250,000 Hz. Recordings lasted 49 ms, with a 5 ms pre-stimulus time, a 24 ms stimulus, and a 20 ms post-stimulus time. Responses were averaged 128 times with stimulus phase alternating on each presentation. The tone burst was initially presented at the highest sound level at each frequency, then reduced in 5 dB steps until the CAP response visually disappeared.

**Fig 2:**
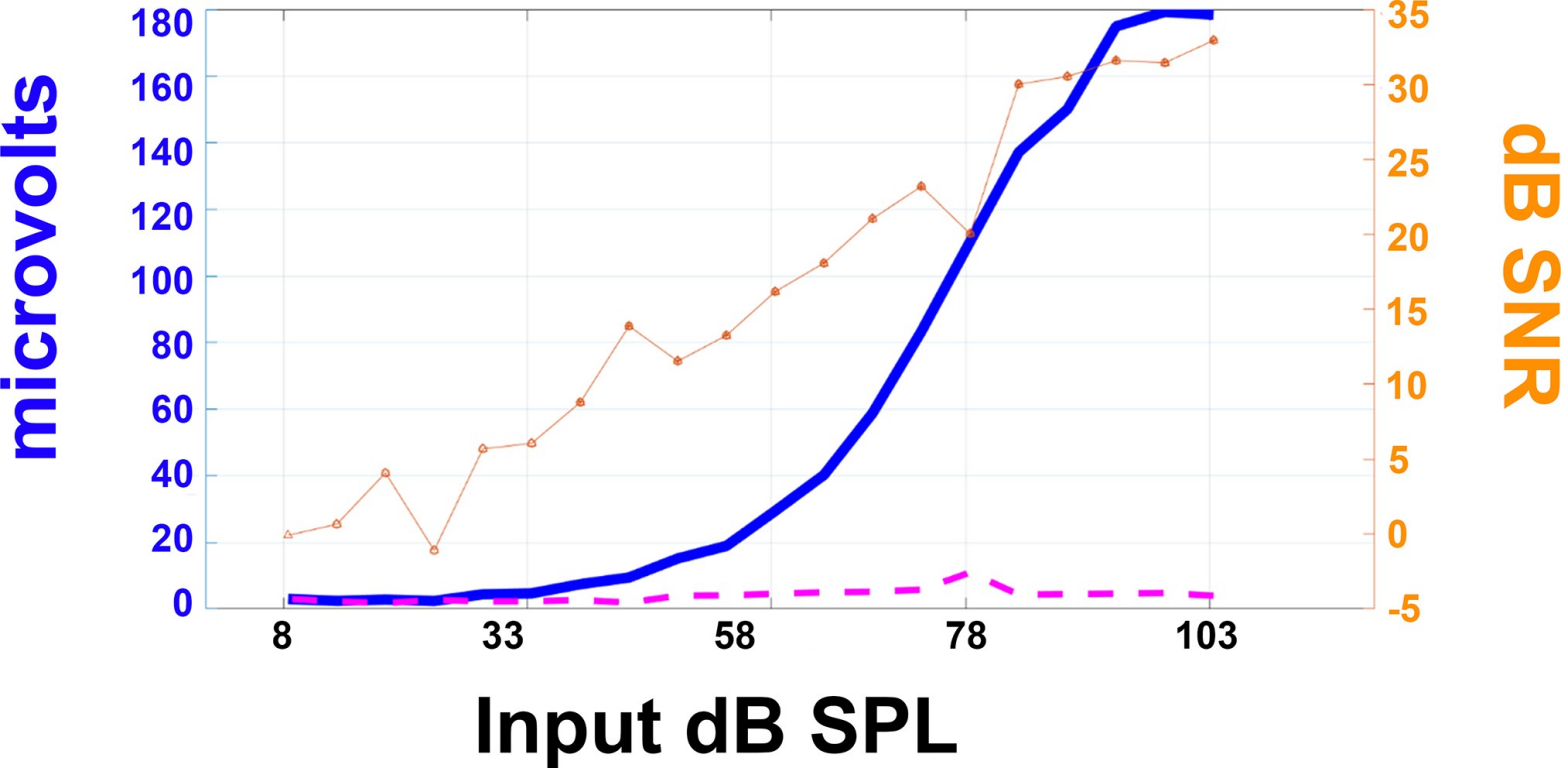
Demonstrates the method used to determine threshold. The bold, solid blue line represents the peak to peak height of the CAP response (in microvolts) to different speaker input levels (dB SPL). The dashed magenta line shows the noise level (microvolts) for the recording taken at each speaker level (dB SPL). The thin, orange line with triangle markers shows the signal to noise ratio in decibels. With a 6 dB threshold definition, the threshold in this example is identified as 28 dB SPL.

CAP thresholds were determined semi-automatically using custom-written MATLAB (MATLAB R2019a) code. To determine the sound level for a visually detectable CAP response (CAP threshold) we compared the peak-to-peak amplitude of the round window recordings between 0–10 ms after the stimulus presentation, the window in which the CAP response occurs, with the peak-to-peak amplitude in a similarly long reference window not containing an auditory response. The threshold was defined as the minimum speaker level eliciting a CAP amplitude at least 3 dB larger than the reference amplitude (Fig 2).

We excluded seven ears after the unsuccessful severing of the ligaments. Each animal and experimental condition’s CAP threshold value can be found in the supplement (S1 Fig). The solid black line gives the mean ± one standard deviation.

### Euthanasia of animals

After the acute physiological experiments, while still under deep anesthesia, the animals were euthanized by a euthasol injection (100–150 mg/kg Pentobarbital) and decapitation. Bullae were then dissected, keeping the middle and inner ears intact. Dissected bullae were kept frozen at -20° C until imaging.

### Imaging

Micro-computed tomography (micro-CT) was performed on five samples using synchrotron radiation at the 2-BM beamline at the Advanced Photon Source at Argonne National Laboratory in Argonne, Illinois. A 25.5 keV monochromatic beam was used. Images were acquired using a 20-micron LuAG scintillator and a CCD-based detector fit with a 2x objective lens. The resolution was 1.7 μm. The field of view was 8 x 1.8 mm. The sample to detector distance was either 120 or 200 mm, depending on the sample. Projections were acquired over 180 degrees of rotation in fly scan mode (continuous sample rotation). Flat and dark field images were captured and used to correct any irregularities in the x-ray beam or the detector during the tomographic reconstruction. A flat field image is an image captured when the x-ray beam is running, but the sample is out of the field of view. A dark field image is an image captured when a shutter blocks the x-ray beam, and the sample is out of the field of view.

Samples were mounted using one of two procedures. In the first setup, bullae were mounted on a scanning stage using clay. A piece of tissue paper moistened with physiological saline solution was placed over the ear canal to maintain the sample’s hydration. The tissue paper was moistened between scans, with scan times being approximately one minute. In the second mounting procedure, each bulla was placed into a plastic tube. Two pieces of 25-micron tungsten wire were placed on the tympanic membrane to assist with sample alignment. A piece of gauze moistened with saline was placed inside the tube along with the bullae to maintain a humid environment to prevent drying of the tympanic membrane and the middle ear.

Tomographic reconstruction was completed with custom code using the open-source TomoPy python package [22]. Reconstructed stacks had 1080 32-bit grayscale images with isotropic voxel size.

For visualization, images were loaded in Fiji/ImageJ at 50% resolution and converted to 8-bit grayscale to reduce the file size [23,24]. Images were then cropped to be large enough to encompass the entire ossicular chain, but small enough to have a file size under 1 GB to perform three-dimensional visualization of image stacks using the ClearVolume plugin [25].

### Data analysis and statistics

This study included four severing conditions (baseline, anterior mallear process severed, posterior incudal ligament severed, and both attachments severed) and seven frequencies tested for each ear for each condition. A two-way ANOVA was run to examine the effect of severing and the interaction between severing and frequency. Statistics were run separately for air and bone conduction thresholds, with only six frequencies measured for bone conduction thresholds. If the p-value for the ANOVA was less than .05, this was followed up by a Tukey-Kramer test to determine which conditions were significantly different from each other.

## Results

### CAP thresholds

**Fig 3:**
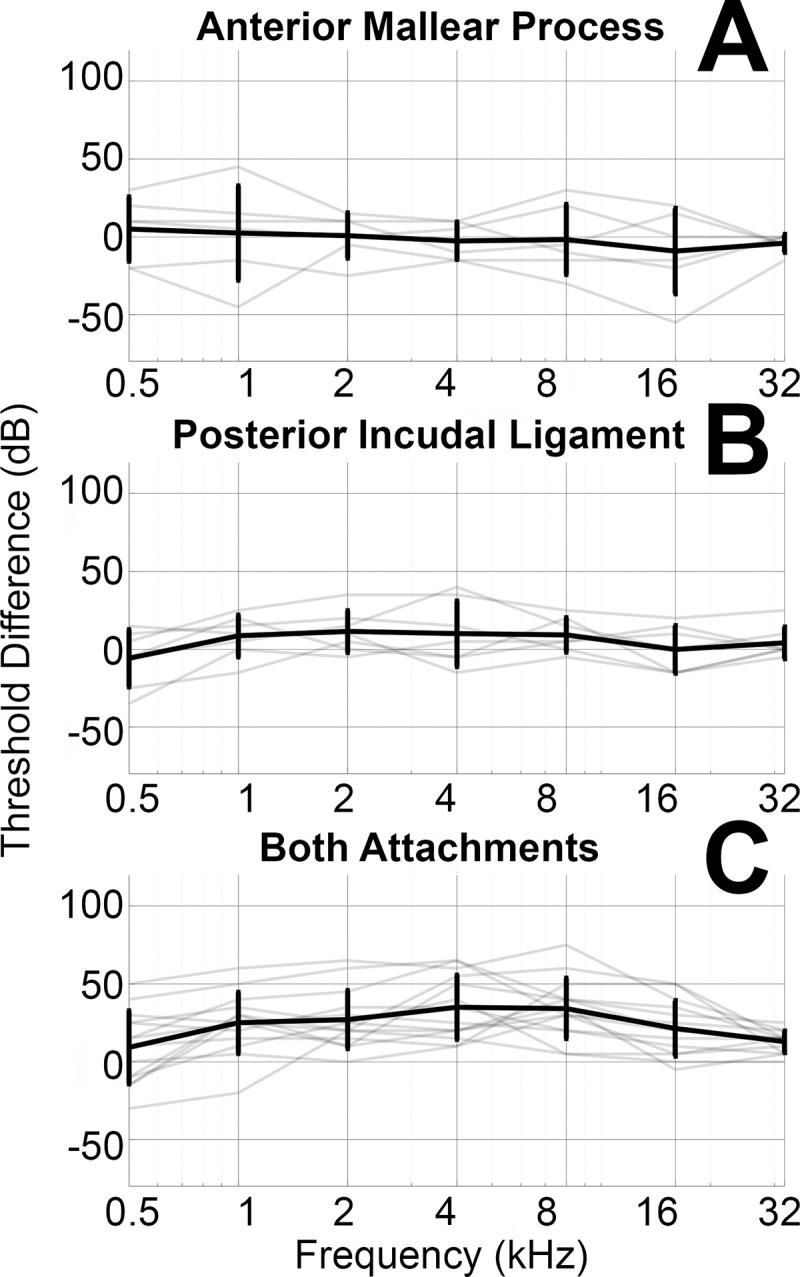
Shows the difference in air conduction thresholds between the anterior mallear process severed condition and baseline (Panel A), the posterior incudal ligament severed condition and baseline (Panel B), and the both attachments severed condition and baseline (Panel C). Grey lines depict threshold differences for individual ears, and the black line depicts mean and standard deviation across ears or each condition. Asterisks indicate a significant difference from other conditions.

Fig 3 shows the air conduction threshold changes resulting from severing one or both middle ear attachments in 17 animals. Data are plotted for each ear, as well as the mean and standard deviation for all ears combined. Between 500–32,000 Hz, CAP threshold differences relative to baseline are -1 dB ± 16 dB with only the anterior mallear process severed (Panel A) and 5dB ± 16 dB with only the posterior incudal ligament severed (Panel B). These differences are not statistically significant. Panel C shows a 23 ± 26 dB CAP threshold shift relative to baseline with both attachments severed. Despite the high variability, the mean threshold shift after both ligaments are severed is significantly greater than the baseline at all frequencies.

For instances where no CAP response was recorded, the maximum speaker output value was used instead. Note that this particularly impacts the CAP thresholds at 32,000 Hz, where the maximum speaker output of 70 dB SPL is within one standard deviation of the mean threshold for all conditions. S2 Table in the supplement displays the number of no-responses recorded for each frequency and condition to clarify the number of ears impacted by this low maximum speaker output.

**Fig 4:**
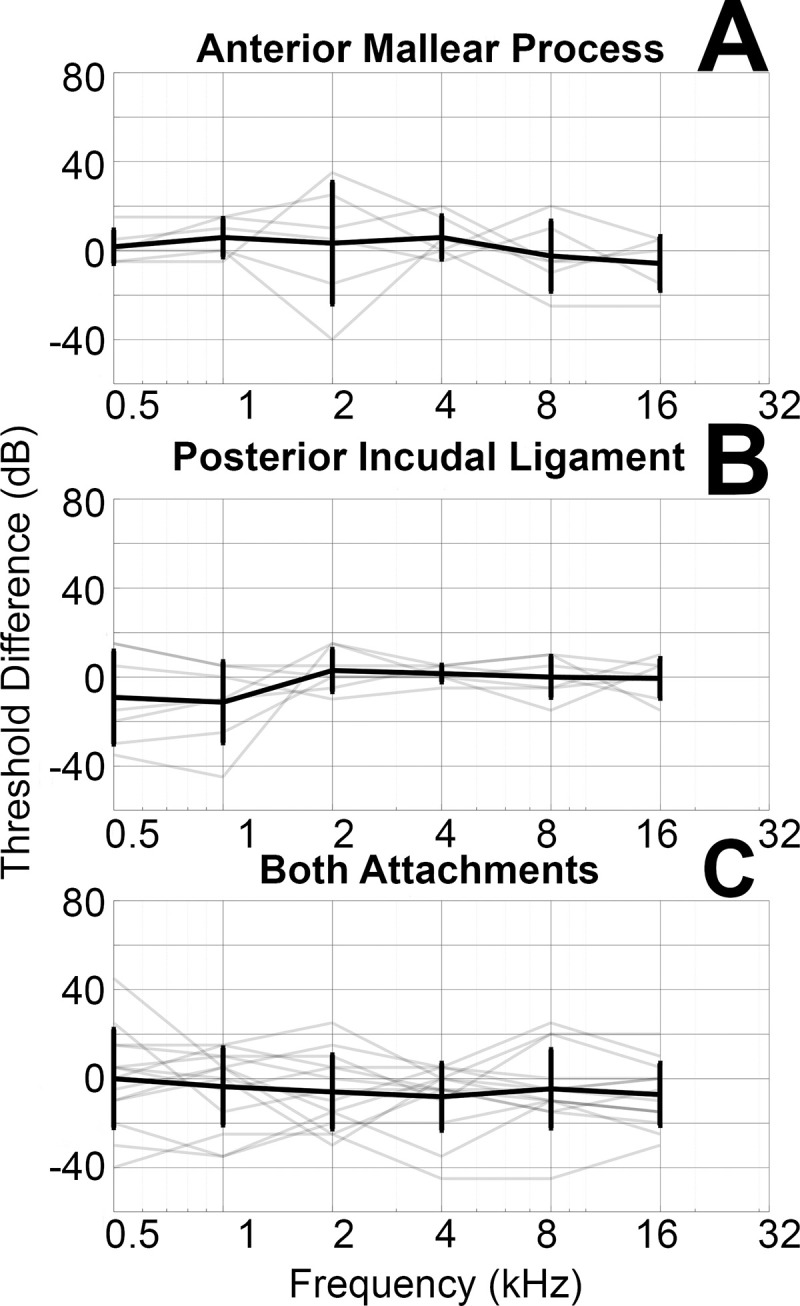
Shows the difference in bone conduction thresholds between the anterior mallear process severed condition and baseline (Panel A), the posterior incudal ligament severed condition and baseline (Panel B), and the both attachments severed condition and baseline (Panel C). Grey lines depict threshold differences for individual ears, and the black line depicts mean and standard deviation across ears or each condition.

Fig 4 shows the bone conduction threshold changes produced by severing one or both middle ear attachments. Threshold values for each condition can be found in the supplement (S2 Fig). Individual ears are shown in gray, while the mean and standard deviation are shown in black. Panel A shows the threshold difference between the baseline condition and the condition with only the anterior mallear process severed. The mean bone conduction threshold difference for frequencies 500–16,000 Hz is 1.3 dB ±7 dB. Panel B displays the threshold difference between the baseline condition and the condition with only the posterior incudal ligament severed. The mean bone conduction threshold difference for frequencies 500–16,000 Hz is -2 dB ± 24 dB. Panel C shows the difference between baseline and the condition with both the anterior mallear process and the posterior incudal ligament severed. The mean bone conduction threshold difference for frequencies 500–16,000 Hz is -4 dB with a standard deviation of ±18 dB. There is no significant difference between bone conduction thresholds for any condition.

**Fig 5:**
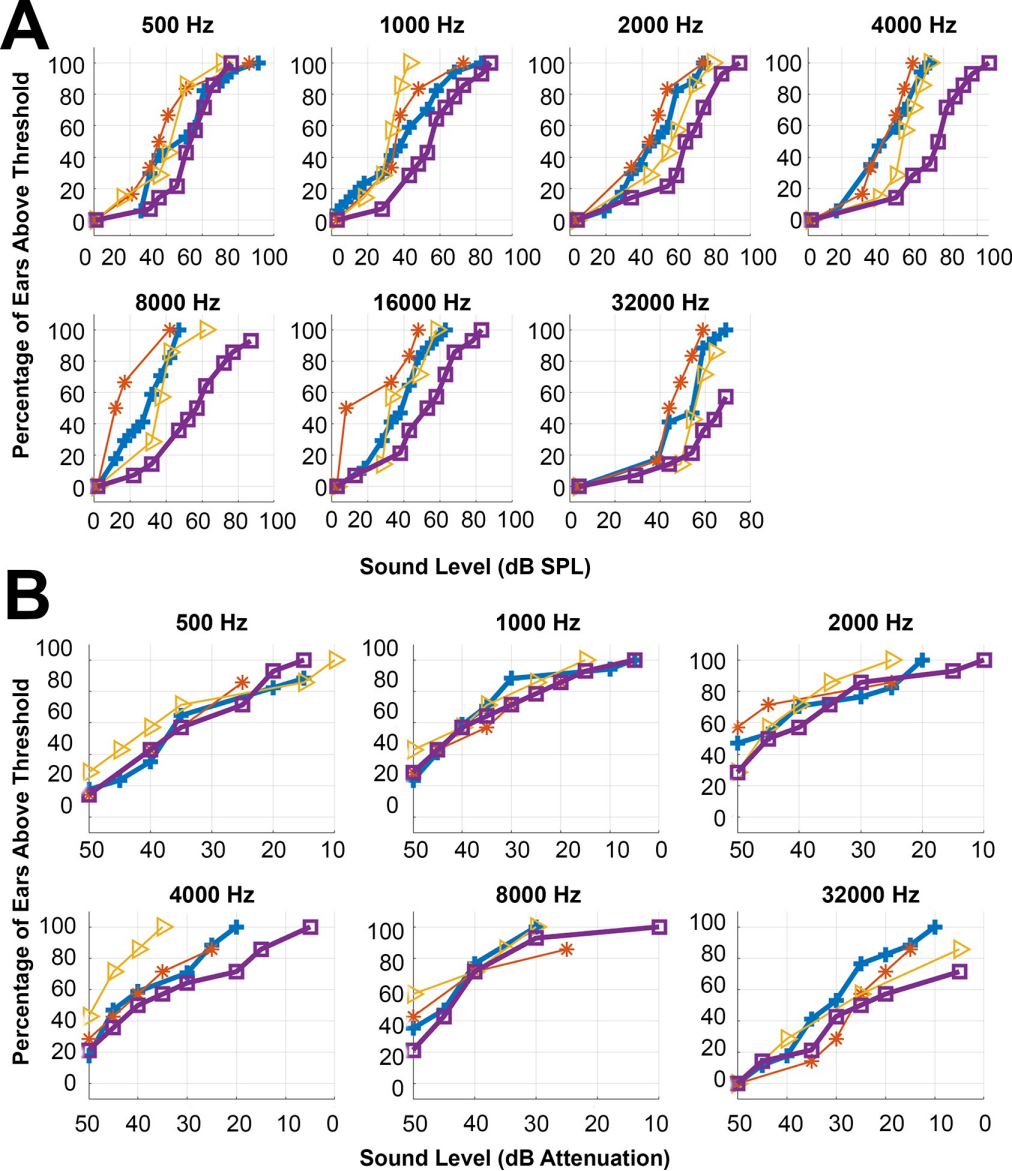
**a** shows cumulative plots of air conduction thresholds at each frequency. The x-axis shows stimulus level in dB SPL. The y-axis shows the percentage of ears in each group that have reached threshold. The baseline condition is blue with plus markers, the anterior mallear process severed condition is orange with asterisk markers, the posterior incudal ligament severed condition is yellow with triangle markers, and the both attachments severed condition is purple with square markers. **b** shows cumulative plots of bone conduction thresholds at each frequency. The x-axis shows stimulus level in dB attenuation, with 0 dB attenuation being the highest input level. The y-axis shows the percentage of ears in each group that have reached threshold. The baseline condition is blue with plus markers, the anterior mallear process severed condition is orange with asterisk markers, the posterior incudal ligament severed condition is yellow with triangle markers, and the both attachments severed condition is purple with square markers.

Fig 5A shows cumulative threshold plots for air conduction thresholds at each frequency. The x-axis shows the intensity of the input stimulus in dB SPL, and the y-axis shows the percentage of ears in each group that have reached the CAP threshold at or before the input stimulus level. The cumulative plot’s rightward shift for the condition with both attachments severed (purple line with square markers) indicates poorer thresholds for that condition. Fig 5B shows cumulative threshold plots for bone conduction thresholds. There is no significant threshold shift for any condition.

The air conduction thresholds for the condition with both attachments severed was significantly different from all other conditions at all frequencies. There were no significant differences between conditions for bone conduction thresholds. For air conduction CAP thresholds, a 2-way ANOVA indicated that there was a significant difference between frequencies (500–32,000 Hz) (F = 9.97, p = 4.64x10^-10^) and attachment status (including baseline, anterior mallear attachment severed, posterior incudal ligament severed, and both attachments severed conditions) (F = 29.79, p = 6.45*10^−17^). Effect size as measured by Cohen’s F was 0.37 for frequencies and 0.50 for attachment status. A Tukey-Kramer post hoc test (S1 Table) revealed that the condition with both attachments severed was significantly different from all other conditions at all frequencies tested. Significant differences between other groups were only found when different frequencies were compared to each other. For bone conduction thresholds, a 2-way ANOVA revealed no significant differences between frequencies (F = 11.9, p = 0) or attachment severing conditions (F = 2.3, p = 0.078). Effect size as measured by Cohen’s F was 0.47 for frequencies and 0.15 for attachment severing conditions.

### Imaging

**Fig 6:**
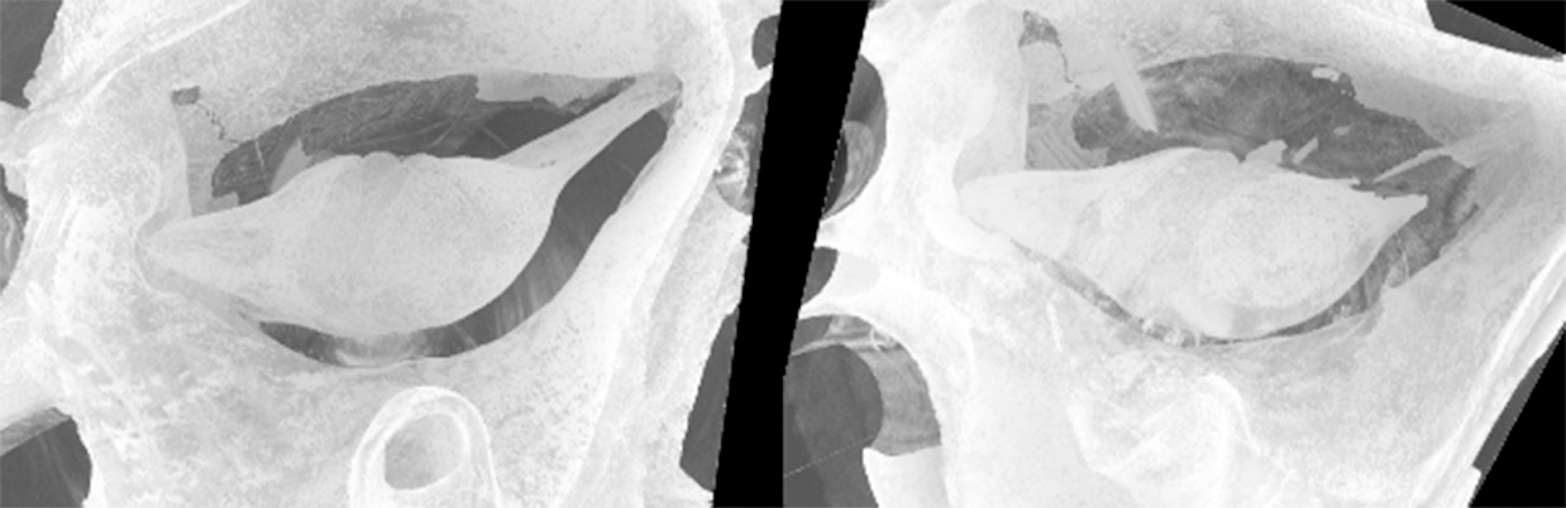
Shows the position of the incus and malleus within the bulla in an ear with intact attachments (left) and an ear with both the posterior incudal ligament and anterior mallear process severed (right). In the intact ear, the incudomallear joint is relatively centered in the bulla. In the ear with severed attachments, the incudomallear joint is displaced, resting towards one side of the cavity.

Fig 6 shows the incus, malleus, and incudomallear joint positions within the middle ear cavity in ears with intact attachments and severed attachments. Qualitatively, the incudomallear joint is centered within the cavity in the ear with intact attachments. In the ear with severed attachments, the incudomallear joint is displaced, resting closer to one side of the cavity.

**Fig 7:**
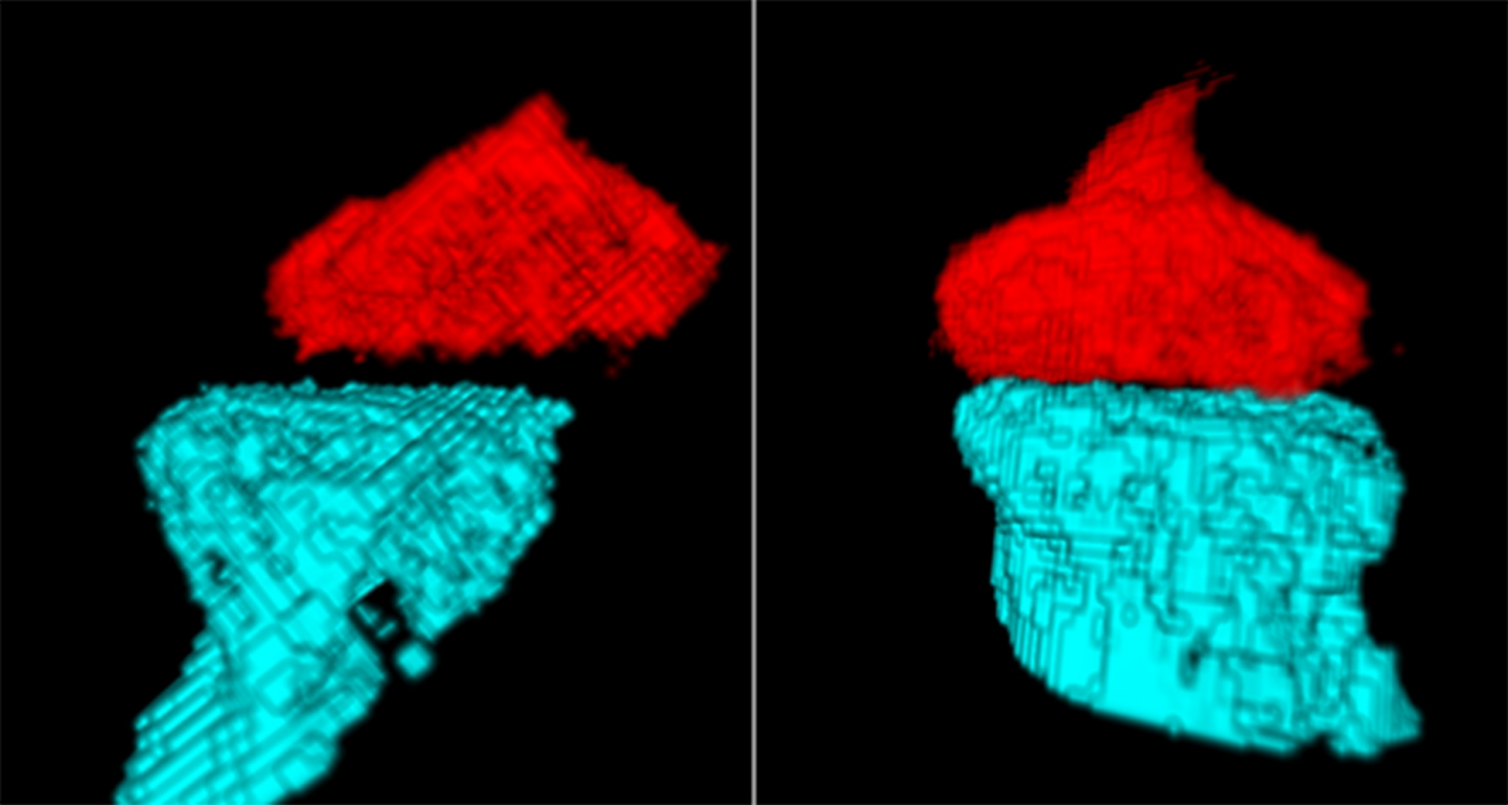
Shows the relative positions of the stapes (blue) and incus (red) at the incudostapedial joint in ears with both attachments severed (left) and both attachments intact (right).

Fig 7 shows the relative positions of the incus and stapes at the incudostapedial joint in ears with intact and severed attachments. Qualitatively, in the ear with intact attachments, the head of the stapes and the lenticular process of the incus sit close together and are well aligned. In the ear with severed attachments, the lenticular process of the incus is displaced from the stapes and misaligned.

## Discussion

The putative role of the ligaments that attach the ossicles to the walls of the middle ear cavity is to suspend the ossicles and define degrees of freedom of vibration [3–12]. This study has shown that cutting both the anterior mallear attachment and the posterior incudal ligament in the gerbil middle ear elevates air conduction thresholds by approximately 20 dB across frequencies, with no significant change in bone conduction thresholds, suggesting that losses are caused by compromised middle ear function. However, cutting one attachment alone does not lead to substantial changes in either air or bone conduction thresholds. These results may suggest redundancy in the middle ear attachments, with one of these two attachments alone being sufficient to maintain normal ossicle position and vibration. The severing of both the posterior incudal ligament and the anterior mallear process appears to alter the ossicular position, with imaging showing changes to the coupling between the incus and the stapes. While the attachments do not play a direct role in sound transmission, the ossicular chain can shift from its optimal position when the attachments are severed, leading to reduced sound transmission. The term “suspensory” to describe these two attachments is consistent with maintaining the position of the ossicles.

Previously published data similarly shows minimal impact for the condition of only one ligament being severed. Selected ligaments were severed individually or in series in human cadaver ears, measuring the change in middle ear transmission with laser doppler vibrometry on the stapes footplate [6,13,14]. No substantial changes in sound transmission were reported in any of these studies. However, none of these studies severed all of the middle ear ligaments together. These results are consistent with our findings, which show that a single attachment may be sufficient to maintain the position of the ossicles, preventing major changes in sound transmission.

Dirckx and Decraemer (2001) [26] severed the stapes, anterior mallear process, and posterior incudal ligament in the gerbil middle ear, studying the response of the tympanic membrane to static pressure changes. In order, the stapes, posterior incudal ligament, and anterior mallear process were severed. Once all of the attachments were severed, substantial changes to the resting position of the tympanic membrane were observed, leading the authors to conclude that the anterior mallear process is essential for maintaining the ossicular position and tympanic membrane tension. In our study, reversing the order of severing the anterior mallear process and the posterior incudal ligament revealed that it is not the anterior mallear process alone, but rather the combination of the anterior mallear process and the posterior incudal ligament that is necessary for maintaining the position of the ossicular chain. This study did not replicate the change in tension of the tympanic membrane after the severing of both attachments, likely because the stapes remained intact, retaining the influence of the annular ligament on maintaining the tension on the ossicular chain and the tympanic membrane.

Elkhouri et al. (2006) [20] modelled the impact of severing the anterior mallear process and posterior incudal ligament in the gerbil ear on sound transmission. In this study, the anterior mallear process was conceptualized as a combination of a long rigid body continuous with the malleus, and a small, ligamentous connection, represented by a spring, at the tip of the anterior mallear process, connecting the malleus to the wall of the middle ear cavity. Severing ligaments was modelled by setting the spring stiffness to zero. Simulating zero stiffness of the anterior mallear ligament or the posterior incudal ligament led to small changes in predicted sound transmission, up to an 18% increase in stapes and umbo vibration for the detachment of the posterior incudal ligament. A large change in axis of rotation was found for the case of an anterior mallear ligament with no stiffness, but minimal change in axis of rotation was predicted for a posterior incudal ligament with no stiffness. The data from our study do not support this model. An increase in stapes and umbo transmission would not be consistent with the poorer CAP thresholds observed in this experiment after both attachments were severed, or the lack of significant change in CAP thresholds with either attachment severed individually. While our study did not examine the axis of rotation directly, the pattern of threshold changes and the qualitative observations of the ossicular chain’s position do not support different impacts on the axis of rotation from severing the anterior mallear process compared to severing the posterior incudal ligament. We do, however, anticipate that there would be a large change in the axis of rotation when both attachments are severed.

Other models of the middle ear that incorporate severing of the attachments have similar limitations to previous experimental studies in that ligaments are only examined individually or in small groups, but never with both the anterior mallear ligament and posterior incudal ligament together [13,21]. Small changes in hearing predicted by these models, less than 10 dB, when ligaments are altered would not be detectable using the CAP method and small sample size used in this study. However, the small threshold changes predicted by these models would not be clinically significant when considering translational applications of this research.

After severing ligaments, fluid collection was frequently observed between the incus and the tympanic membrane or between the incus and the wall of the bulla on the medial side of the incus. The ossicles were observed to become more mobile if the fluid bonds were disrupted by sweeping a 25-μm tungsten wire between the two fluid sites. This raises the question of whether these fluid bonds maintain the position of the ossicles during the experiment, preventing a systematic change in threshold for conditions with only one attachment severed. In a more realistic context where an animal’s head is moving and the ear is exposed to sizeable static pressure changes, the fluid bonds may be more susceptible to disruption than intact ligaments, leading to differences not detected in this experiment. Additionally, the way that the ossicles settle after severing, and the location and extent of fluid bonds formed is not something that can be controlled for. Thus, the condition with both attachments severed, rather than representing one configuration, may contain a number of configurations with differing fluid bonds and thus resting states of the ossicles. This heterogeneity likely contributes to the variability of hearing thresholds in the final condition.

It is worth noting that the condition where both attachments are severed is always measured last, regardless of the order of severing. This means that CAPs for this condition are measured after the animal has been under isoflurane anesthesia for several hours. This leads to the possibility that a systematic sensorineural hearing loss induced by prolonged anesthesia may have influenced results for this condition. However, a systematic sensorineural component would result in poorer thresholds in response to bone conduction stimulation for the condition with both attachments severed compared to baseline. No significant change in bone conduction thresholds between the condition with both attachments severed and baseline supports the hypothesis that changes in hearing in response to air conduction stimuli represent changes in middle ear function.

A limitation of translating this study to human ears is that the gerbil anterior mallear process is a very thin, translucent bone. In contrast, the human anterior mallear ligament is a true ligament. Other animals commonly used in middle ear research have more divergent anatomy, such as the thicker anterior mallear process in the mouse and the absence of an analogous structure in the guinea pig [10,15]. The bony anterior process is considered to perform analogously to the human ligament, providing a rotational axis [10,12]. In this case, it is important to keep in mind that a bony connection is not a fixed connection, particularly due to the bone’s thinness and the very small amplitude of the ossicular vibrations.

## Conclusion

In this study, the anterior mallear attachment and the posterior incudal ligament of the gerbil middle ear were severed. Changes in hearing were measured via air conduction and bone conduction compound action potential recordings. Air conduction thresholds were significantly elevated from baseline when both attachments were severed, but not when each attachment was severed individually. Bone conduction thresholds did not significantly differ from baseline in any condition. The conductive hearing loss resulting from the severing of both middle ear attachments supports the hypothesis that these attachments play an essential role in sound transmission through the middle ear. This has implications for the future development of middle ear prostheses, particularly as advances in 3D printing make the possibility of a three-ossicle prosthesis more feasible [1]. In developing a three-ossicle prosthesis, a component to recreate the function of middle ear ligaments is seemingly important for optimal sound transmission.

## Supporting information

S1 Fig Air conduction thresholds for each frequency in dB SPL. Light grey is individual ears. Solid black is the mean and standard deviation. Dashed black is the maximum output of the speaker. **A** shows thresholds for the baseline condition, before attachments are severed (17 ears). **B** shows thresholds for ears with only the anterior mallear process severed (7 ears). **C** shows thresholds for ears with only the posterior incudal ligament severed (7 ears). **D** shows thresholds for ears with both attachments severed (14 ears).
(TIF)

S2 Fig Bone conduction thresholds for each frequency in dB attenuation. Light grey is individual ears. Solid black is the mean and standard deviation. Dashed black is the maximum output of the bone conduction transducer. **A** shows thresholds for the baseline condition, before attachments are severed (17 ears). **B** shows thresholds for ears with only the anterior mallear process severed (7 ears). **C** shows thresholds for ears with only the posterior incudal ligament severed (7 ears). **D** shows thresholds for ears with both attachments severed (14 ears).
(TIF)

S3 Fig Shows the mean and standard deviation of thresholds for each condition. **A** shows air-conduction thresholds in dB SPL. **B** shows bone-conduction thresholds in dB attenuation. On both figures, Baseline condition is blue with cross markers, Anterior Mallear Process Severed condition is orange with square markers, Posterior Incudal Ligament Severed condition is yellow with triangle markers, and Both Attachments Severed condition is purple with asterisk markers. The dashed black line indicates the maximum transducer output at each frequency. Background highlighting indicates the frequency ranges where thresholds are averaged to analyze low (blue), mid (green), and high (magenta) frequency regions.
(TIF)

S1 Table Results of a Tukey-Kramer test comparing attachment severing condition and frequency for air conduction thresholds. Attachment severing conditions include baseline (bl), anterior mallear process severed (MP), posterior incudal ligament severed (IL), and both attachments severed (MPIL). Frequencies are listed in kHz. Each box contains the p-value for the comparison of the intersecting conditions. P-values less than .05 have a green background fill. P-values greater than .05 have a blue background fill. The dark green background fill denotes a significant comparison between two different attachment severing conditions at the same frequency.
(DOCX)

S2 Table Number of no response thresholds, displayed by condition, stimulation method, and frequency (Hz). Thresholds were not measured at 32000 Hz via bone conduction stimulation due to frequency limitations of the transducer.
(DOCX)

## References

[1] Hirsch JD, Vincent RL, Eisenman DJ. Surgical reconstruction of the ossicular chain with custom 3D printed ossicular prosthesis. 3D Print Med. 2017;3: 4–11. doi: 10.1186/s41205-017-0012-5 29782607 PMC5954796

[2] Kamrava B, Gerstenhaber JA, Amin M, Har-El Y El, Roehm PC. Preliminary Model for the Design of a Custom Middle Ear Prosthesis. Otol Neurotol. 2017;38: 839–845. doi: 10.1097/MAO.0000000000001403 28441229

[3] Ludwig C. Lehrbuch der Physiologie des Menschen. 1852.

[4] Anatomy Gray H., Descriptive and Surgical. 1878.

[5] Decraemer WF, Khanna SM. Modelling the malleus vibration as a rigid body motion with one rotational and one translational degree of freedom. Hear Res. 1994;72: 1–18. doi: 10.1016/0378-5955(94)90199-6 8150727

[6] Gan RZ, Cheng T, Nakmali D, Wood MW. Effects of Middle Ear Suspensory Ligaments on Acoustic-Mechanical Transmission in Human Ear. Middle Ear Mech Res Otol. 2007; 212–221. doi: 10.1142/9789812708694_0029

[7] Lemmerling MM, Stambuk HE, Mancuso AA, Antonelli PJ, Kubilis PS. CT of the normal suspensory ligaments of the ossicles in the middle ear. Am J Neuroradiol. 1997;18: 471–477. 9090405 PMC8338412

[8] Sim JH, Puria S. Soft tissue morphometry of the malleus-incus complex from micro-CT imaging. JARO—J Assoc Res Otolaryngol. 2008;9: 5–21. doi: 10.1007/s10162-007-0103-x 18311579 PMC2536804

[9] Lavender D, Taraskin SN, Mason MJ. Mass distribution and rotational inertia of “microtype” and “freely mobile” middle ear ossicles in rodents. Hear Res. 2011;282: 97–107. doi: 10.1016/j.heares.2011.09.003 21951489

[10] Mason MJ. Of mice, moles and guinea pigs: Functional morphology of the middle ear in living mammals. Hear Res. 2013;301: 4–18. doi: 10.1016/j.heares.2012.10.004 23099208

[11] Maftoon N, Funnell WRJ, Daniel SJ, Decraemer WF. Finite - Element Modelling of the Response of the Gerbil Middle Ear to Sound. J Assoc Res Otolaryngol. 2015;16: 547–567. doi: 10.1007/s10162-015-0531-y 26197870 PMC4569606

[12] Fleischer G. Evolutionary principles of the mammalian middle ear. Adv Anta Embryol Cell Biol. 1978;55: 1–70. doi: 10.1007/978-3-642-67143-2 735912

[13] Dai C, Cheng T, Wood MW, Gan RZ. Fixation and detachment of superior and anterior malleolar ligaments in human middle ear: Experiment and modeling. Hear Res. 2007;230: 24–33. doi: 10.1016/j.heares.2007.03.006 17517484 PMC2039917

[14] Hato N, Welsh JT, Goode RL, Stenfelt S. Acoustic role of the buttress and posterior incudal ligament in human temporal bones. Otolaryngol—Head Neck Surg. 2001;124: 274–278. doi: 10.1067/mhn.2001.113664 11240990

[15] Kobayashi M. On the ligaments and articulations of the auditory ossicles of rat and guinea pig. Hiroshima J Med Sci. 1955;3: 343–351.

[16] Kobayashi M. On the ligaments and articulations of the auditory ossicles of cow, swine and goat. Hiroshima J Med Sci. 1955;3: 331–342.

[17] Mason MJ, Farr MRB. Flexibility within the middle ears of vertebrates. J Laryngol Otol. 2013;127: 2–14. doi: 10.1017/S0022215112002496 23146175

[18] De Greef D, Buytaert JAN, Aerts JRM, Van Hoorebeke L, Dierick M, Dirckx J. Details of human middle ear morphology based on micro-CT imaging of phosphotungstic acid stained samples. J Morphol. 2015;276: 1025–46. doi: 10.1002/jmor.20392 26010747

[19] Mansour S, Magnan J, Haidar H, Nicolas K, Louryan S. Comprehensive and Clinical Anatomy of the Middle Ear. Springer; 2013. doi: 10.1007/978-3-642-36967-4

[20] Elkhouri N, Liu H, Funnell WRJ. Low-frequency finite-element modeling of the gerbil middle ear. JARO—J Assoc Res Otolaryngol. 2006;7: 399–411. doi: 10.1007/s10162-006-0055-6 17043944 PMC2504629

[21] Calero D, Lobato L, Paul S, Cordioli JA. Analysis of the human middle ear dynamics through multibody modeling. J Biomech Eng. 2020;142. doi: 10.1115/1.4046689 32191261

[22] Gürsoy D, De Carlo F, Xiao X, Jacobsen C. TomoPy: A framework for the analysis of synchrotron tomographic data. J Synchrotron Radiat. 2014;21: 1188–1193. doi: 10.1107/S1600577514013939 25178011 PMC4181643

[23] Schindelin J, Arganda-Carreras I, Frise E, Kaynig V, Longair M, Pietzsch T, et al. Fiji: an open-source platform for biological-image analysis. Nat Methods. 2012;9: 676–682. doi: 10.1038/nmeth.2019 22743772 PMC3855844

[24] Schneider C a Rasband WS, Eliceiri KW. NIH Image to ImageJ: 25 years of image analysis. Nat Methods. 2012;9: 671–675. doi: 10.1038/nmeth.2089 22930834 PMC5554542

[25] Royer LA, Weigert M, Günther U, Maghelli N, Jug F, Sbalzarini IF, et al. ClearVolume: Open-source live 3D visualization for light-sheet microscopy. Nat Methods. 2015;12: 480–481. doi: 10.1038/nmeth.3372 26020498

[26] Dirckx JJJ, Decraemer WF. Effect of middle ear components on eardrum quasi-static deformation. Hear Res. 2001;157 (1–2): 124–137. doi: 10.1016/s0378-5955(01)00290-8 11470192

