# Supplementary figures and images for: Are suspensory ligaments important for middle ear reconstruction?
Source: PLoS One. 2021 Aug 24;16(8):e0255821. doi: 10.1371/journal.pone.0255821 (PMC8384183; doi:10.1371/journal.pone.0255821)

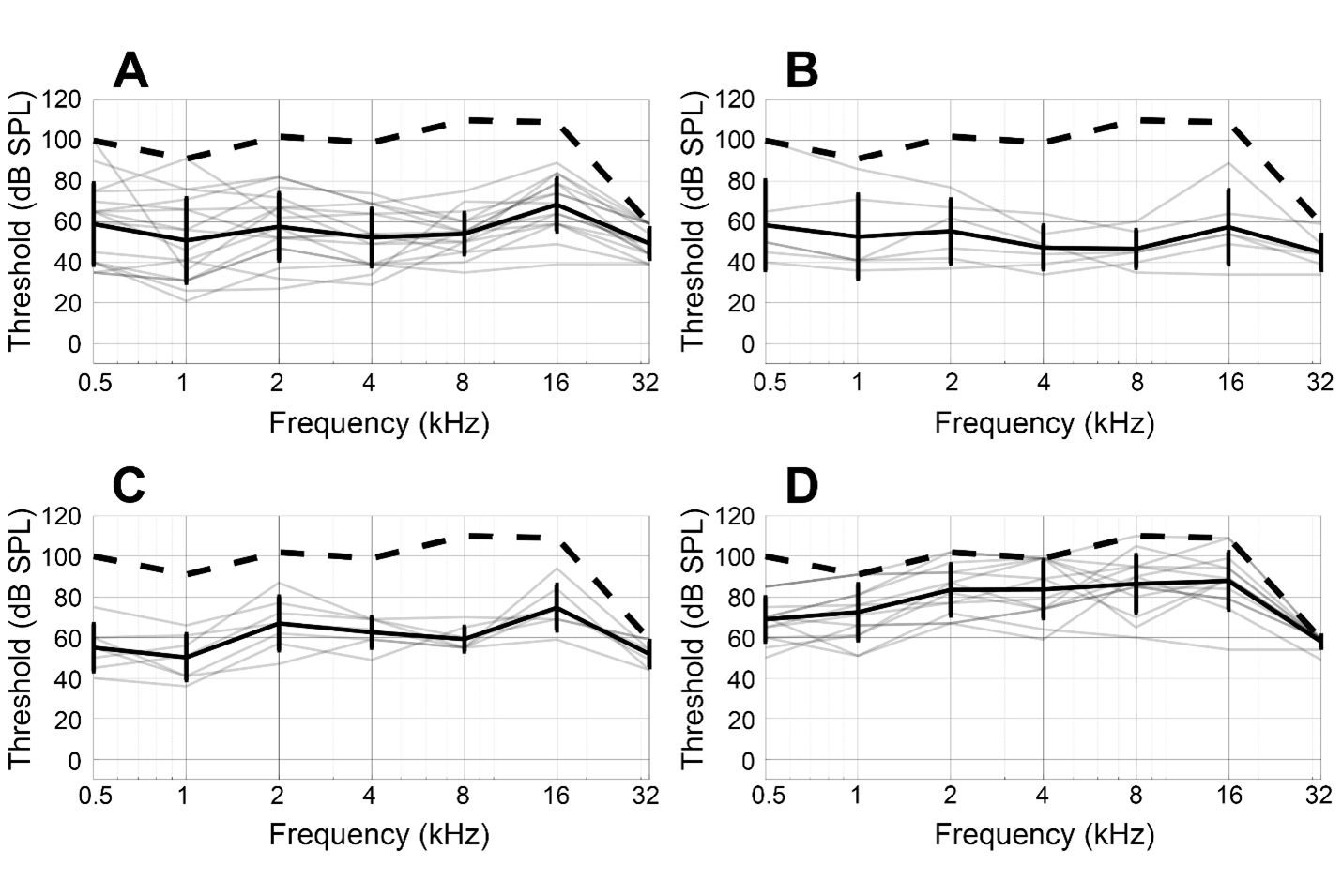

Supplement: S1 Fig — Light grey is individual ears. Solid black is the mean and standard deviation. Dashed black is the maximum output of the speaker. A shows thresholds for the baseline condition, before attachments are severed (17 ears). B shows thresholds for ears with only the anterior mallear process severed (7 ears). C shows thresholds for ears with only the posterior incudal ligament severed (7 ears). D shows thresholds for ears with both attachments severed (14 ears). (TIF) [file pone.0255821.s001.tif]

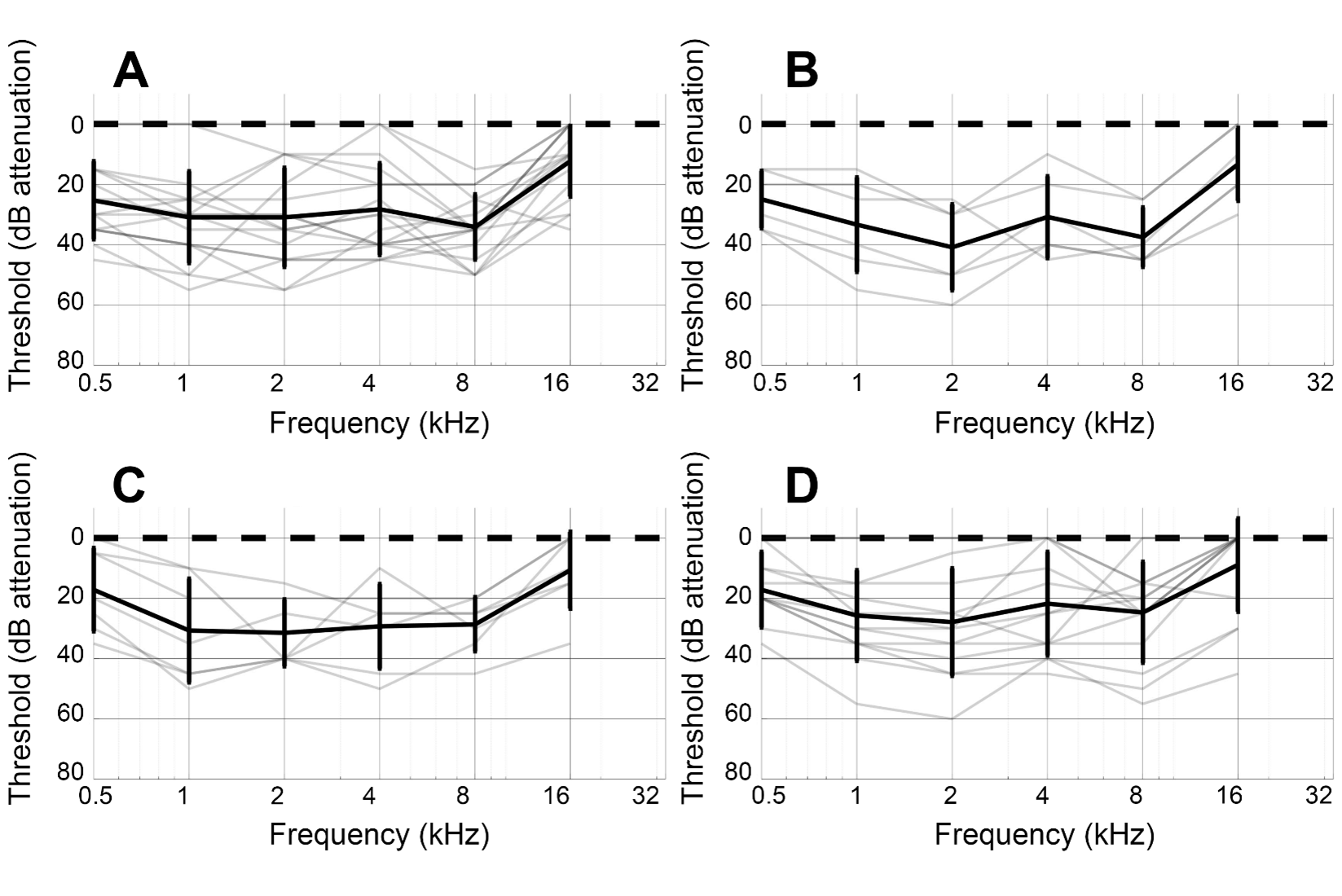

Supplement: S2 Fig — Light grey is individual ears. Solid black is the mean and standard deviation. Dashed black is the maximum output of the bone conduction transducer. A shows thresholds for the baseline condition, before attachments are severed (17 ears). B shows thresholds for ears with only the anterior mallear process severed (7 ears). C shows thresholds for ears with only the posterior incudal ligament severed (7 ears). D shows thresholds for ears with both attachments severed (14 ears). (TIF) [file pone.0255821.s002.tif]

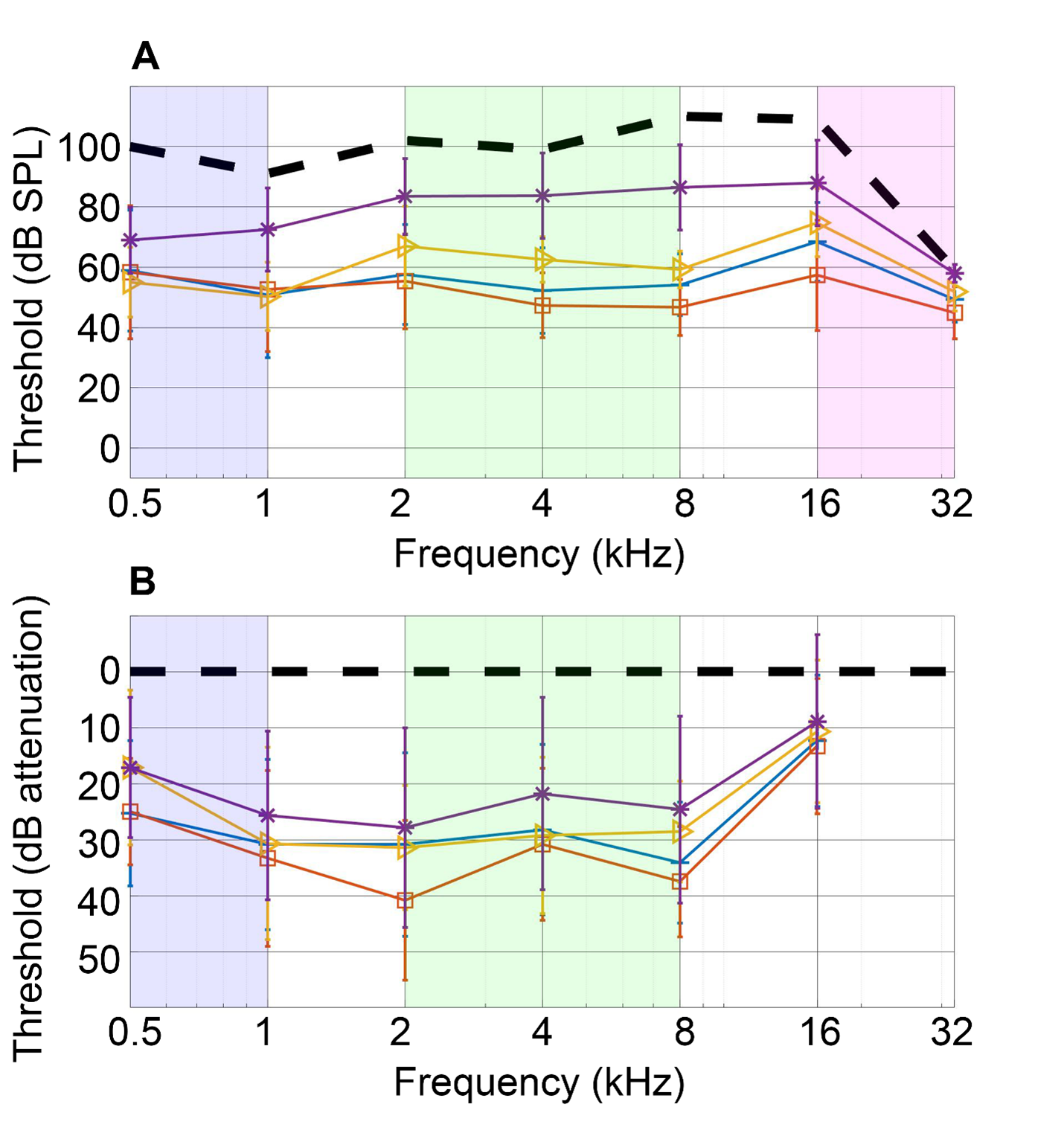

Supplement: S3 Fig — A shows air-conduction thresholds in dB SPL. B shows bone-conduction thresholds in dB attenuation. On both figures, Baseline condition is blue with cross markers, Anterior Mallear Process Severed condition is orange with square markers, Posterior Incudal Ligament Severed condition is yellow with triangle markers, and Both Attachments Severed condition is purple with asterisk markers. The dashed black line indicates the maximum transducer output at each frequency. Background highlighting indicates the frequency ranges where thresholds are averaged to analyze low (blue), mid (green), and high (magenta) frequency regions. (TIF) [file pone.0255821.s003.tif]
