# Supplementary material for: Are suspensory ligaments important for middle ear reconstruction?
Source: PLoS One. 2021 Aug 24;16(8):e0255821. doi: 10.1371/journal.pone.0255821 (PMC8384183; doi:10.1371/journal.pone.0255821)
Supplement: S1 Table — Attachment severing conditions include baseline (bl), anterior mallear process severed (MP), posterior incudal ligament severed (IL), and both attachments severed (MPIL). Frequencies are listed in kHz. Each box contains the p-value for the comparison of the intersecting conditions. P-values less than .05 have a green background fill. P-values greater than .05 have a blue background fill. The dark green background fill denotes a significant comparison between two different attachment severing conditions at the same frequency. (DOCX) [file pone.0255821.s004.docx]

|  | bl 1 | bl 2 | bl 4 | bl 8 | bl 16 | bl 32 | MP0.5 | MP1 | MP2 | MP4 | MP8 | MP16 | MP32 | IL0.5 | IL1 | IL2 | IL4 | IL8 | IL16 | IL32 | MPIL0.5 | MPIL1 | MPIL2 | MPIL4 | MPIL8 | MPIL16 | MPIL32 |
| --- | --- | --- | --- | --- | --- | --- | --- | --- | --- | --- | --- | --- | --- | --- | --- | --- | --- | --- | --- | --- | --- | --- | --- | --- | --- | --- | --- |
| bl 0.5 | 1.0 | 1.0 | 0.5 | 0.3 | 0.8 | 1.0 | 1.0 | 0.9 | 1.0 | 1.0 | 0.1 | 0.4 | 1.0 | 1.0 | 1.0 | 0.9 | 0.3 | 1.0 | 1.0 | 0.9 | 2.0E-06 | 0.3 | 4.5E-06 | 2.0E-06 | 0.9 | 0.8 | 3.8E-06 |
| bl 1 |  | 0.2 | 0.01 | 1.0 | 1.0 | 0.1 | 1.0 | 1.0 | 1.0 | 0.9 | 0.9 | 1.0 | 1.0 | 0.9 | 1.0 | 0.1 | 0.01 | 1.0 | 1.0 | 0.1 | 7.9E-06 | 2.0E-06 | 2.0E-06 | 2.0E-06 | 0.2 | 0.06 | 2.0E-06 |
| bl 2 |  |  | 1.0 | 0.0 | 0.0 | 1.0 | 0.8 | 0.1 | 1.0 | 1.0 | 1.5E-03 | 0.0 | 1.0 | 1.0 | 1.0 | 1.0 | 1.0 | 0.5 | 0.8 | 1.0 | 0.4 | 1.0 | 2.0E-06 | 6.5E-05 | 1.0 | 1.0 | 3.3E-03 |
| bl 4 |  |  |  | 6.8E-06 | 0.00 | 1.0 | 0.2 | 0.01 | 0.9 | 1.0 | 3.2E-05 | 3.3E-04 | 0.9 | 1.0 | 0.7 | 1.0 | 1.0 | 0.08 | 0.2 | 1.0 | 0.9 | 1.0 | 0.1 | 2.0E-06 | 1.0 | 1.0 | 0.09 |
| bl 8 |  |  |  |  | 1.0 | 0.00 | 1.0 | 1.0 | 0.7 | 0.1 | 1.0 | 1.0 | 0.7 | 0.2 | 0.9 | 2.7E-03 | 5.4E-05 | 1.0 | 1.0 | 2.2E-03 | 2.0E-06 | 1.1E-05 | 2.0E-06 | 2.0E-06 | 2.0E-06 | 4.1E-04 | 2.0E-06 |
| bl 16 |  |  |  |  |  | 0.01 | 1.0 | 1.0 | 0.9 | 0.4 | 1.0 | 1.0 | 0.9 | 0.6 | 1.0 | 0.02 | 5.8E-04 | 1.0 | 1.0 | 0.02 | 2.0E-06 | 1.5E-04 | 2.0E-06 | 2.0E-06 | 0.03 | 2.0E-06 | 2.0E-06 |
| bl 32 |  |  |  |  |  |  | 0.8 | 0.09 | 1.0 | 1.0 | 1.2E-03 | 9.2E-03 | 1.0 | 1.0 | 1.0 | 1.0 | 1.0 | 0.4 | 0.8 | 1.0 | 0.4 | 1.0 | 5.3E-03 | 8.6E-05 | 1.0 | 1.0 | 2.0E-06 |
| MP0.5 |  |  |  |  |  |  |  | 1.0 | 1.0 | 0.5 | 0.3 | 0.8 | 1.0 | 0.7 | 1.0 | 0.4 | 0.06 | 1.0 | 1.0 | 0.3 | 2.0E-06 | 0.04 | 2.2E-06 | 2.0E-06 | 0.6 | 0.2 | 2.1E-06 |
| MP1 |  |  |  |  |  |  |  |  | 0.2 | 0.01 | 1.0 | 1.0 | 0.1 | 0.5 | 0.7 | 0.02 | 7.7E-04 | 1.0 | 1.0 | 0.02 | 2.4E-06 | 2.0E-06 | 2.0E-06 | 2.0E-06 | 0.03 | 5.3E-03 | 2.0E-06 |
| MP2 |  |  |  |  |  |  |  |  |  | 1.0 | 1.1E-03 | 0.01 | 1.0 | 1.0 | 1.0 | 0.7 | 0.7 | 1.0 | 1.0 | 0.9 | 0.06 | 0.7 | 2.0E-06 | 6.1E-06 | 1.0 | 0.9 | 2.3E-04 |
| MP4 |  |  |  |  |  |  |  |  |  |  | 6.8E-06 | 1.3E-04 | 1.0 | 1.0 | 1.0 | 1.0 | 0.7 | 0.8 | 0.9 | 1.0 | 0.4 | 1.0 | 0.01 | 2.0E-06 | 1.0 | 1.0 | 8.2E-03 |
| MP8 |  |  |  |  |  |  |  |  |  |  |  | 1.0 | 8.3E-04 | 0.04 | 0.5 | 1.9E-04 | 5.3E-06 | 0.7 | 0.9 | 1.5E-04 | 2.0E-06 | 2.6E-06 | 2.0E-06 | 2.0E-06 | 2.0E-06 | 2.9E-05 | 2.0E-06 |
| MP16 |  |  |  |  |  |  |  |  |  |  |  |  | 0.01 | 0.1 | 0.8 | 1.5E-03 | 3.9E-05 | 1.0 | 0.7 | 1.2E-03 | 2.0E-06 | 1.2E-05 | 2.0E-06 | 2.0E-06 | 2.5E-03 | 2.0E-06 | 2.0E-06 |
| MP32 |  |  |  |  |  |  |  |  |  |  |  |  |  | 1.0 | 1.0 | 1.0 | 0.8 | 1.0 | 1.0 | 0.7 | 0.0 | 0.7 | 3.7E-04 | 7.5E-06 | 1.0 | 0.9 | 2.0E-06 |
| IL0.5 |  |  |  |  |  |  |  |  |  |  |  |  |  |  | 1.0 | 1.0 | 0.5 | 0.3 | 0.8 | 1.0 | 2.2E-04 | 0.9 | 2.5E-03 | 5.2E-05 | 1.0 | 1.0 | 2.0E-03 |
| IL1 |  |  |  |  |  |  |  |  |  |  |  |  |  |  |  | 0.2 | 6.6E-03 | 1.0 | 1.0 | 0.1 | 4.5E-03 | 2.2E-04 | 7.7E-06 | 2.0E-06 | 0.9 | 0.7 | 6.2E-06 |
| IL2 |  |  |  |  |  |  |  |  |  |  |  |  |  |  |  |  | 1.0 | 1.1E-03 | 0.01 | 1.0 | 0.9 | 1.0 | 2.2E-04 | 0.02 | 1.0 | 1.0 | 0.2 |
| IL4 |  |  |  |  |  |  |  |  |  |  |  |  |  |  |  |  |  | 6.8E-06 | 1.3E-04 | 1.0 | 1.0 | 1.0 | 0.8 | 2.2E-04 | 1.0 | 1.0 | 0.8 |
| IL8 |  |  |  |  |  |  |  |  |  |  |  |  |  |  |  |  |  |  | 1.0 | 8.3E-04 | 2.0E-05 | 6.2E-03 | 2.0E-06 | 2.0E-06 | 2.2E-04 | 0.07 | 2.0E-06 |
| IL16 |  |  |  |  |  |  |  |  |  |  |  |  |  |  |  |  |  |  |  | 0.01 | 2.2E-04 | 0.04 | 2.1E-06 | 2.0E-06 | 0.6 | 2.2E-04 | 2.0E-06 |
| IL32 |  |  |  |  |  |  |  |  |  |  |  |  |  |  |  |  |  |  |  |  | 0.9 | 1.0 | 0.2 | 0.03 | 1.0 | 1.0 | 2.2E-04 |
| MPIL0.5 | |  |  |  |  |  |  |  |  |  |  |  |  |  |  |  |  |  |  |  |  | 1.0 | 1.0 | 0.5 | 0.3 | 0.8 | 1.0 |
| MPIL1 | |  |  |  |  |  |  |  |  |  |  |  |  |  |  |  |  |  |  |  |  |  | 0.2 | 6.6E-03 | 1.0 | 1.0 | 0.1 |
| MPIL2 | |  |  |  |  |  |  |  |  |  |  |  |  |  |  |  |  |  |  |  |  |  |  | 1.0 | 1.1E-03 | 0.01 | 1.0 |
| MPIL4 | |  |  |  |  |  |  |  |  |  |  |  |  |  |  |  |  |  |  |  |  |  |  |  | 6.8E-06 | 1.3E-04 | 1.0 |
| MPIL8 | |  |  |  |  |  |  |  |  |  |  |  |  |  |  |  |  |  |  |  |  |  |  |  |  | 1.0 | 8.3E-04 |
| MPIL16 | |  |  |  |  |  |  |  |  |  |  |  |  |  |  |  |  |  |  |  |  |  |  |  |  |  | 0.01 |
| **Table S.1:** Results of a Tukey-Kramer test comparing attachment severing condition and frequency for air conduction thresholds. Attachment severing conditions include baseline (bl), anterior mallear process severed (MP), posterior incudal ligament severed (IL), and both attachments severed (MPIL). Frequencies are listed in kHz. Each box contains the p-value for the comparison of the intersecting conditions. P-values less than .05 have a green background fill. P-values greater than .05 have a blue background fill. The dark green background fill denotes a significant comparison between two different attachment severing conditions at the same frequency. | | | | | | | | | | | | | | | | | | | | | | | | | | | |
