# Supplementary material for: Are suspensory ligaments important for middle ear reconstruction?
Source: PLoS One. 2021 Aug 24;16(8):e0255821. doi: 10.1371/journal.pone.0255821 (PMC8384183; doi:10.1371/journal.pone.0255821)
Supplement: S2 Table — Thresholds were not measured at 32000 Hz via bone conduction stimulation due to frequency limitations of the transducer. (DOCX) [file pone.0255821.s005.docx]

|  | 500 | 1000 | 2000 | 4000 | 8000 | 16000 | 32000 |
| --- | --- | --- | --- | --- | --- | --- | --- |
| Air Conduction  Baseline | 1 | 0 | 0 | 0 | 0 | 0 | 4 |
| Air Conduction  AMP Severed | 0 | 0 | 0 | 0 | 0 | 0 | 0 |
| Air Conduction  PIL Severed | 0 | 0 | 0 | 0 | 0 | 0 | 2 |
| Air Conduction  AMP & PIL Severed | 0 | 3 | 2 | 3 | 1 | 2 | 12 |
| Bone Conduction  Baseline | 2 | 2 | 0 | 0 | 0 | 5 | - |
| Bone Conduction  AMP Severed | 0 | 0 | 0 | 0 | 1 | 0 | - |
| Bone Conduction  PIL Severed | 1 | 0 | 0 | 0 | 0 | 3 | - |
| Bone Conduction  AMP & PIL Severed | 2 | 2 | 2 | 3 | 2 | 9 | - |
| **Table S.2:** Number of no response thresholds, displayed by condition, stimulation method, and frequency (Hz). Thresholds were not measured at 32000 Hz via bone conduction stimulation due to frequency limitations of the transducer. | | | | | | | |
